# Brazilian Spotted Fever with an Approach in Veterinary Medicine and One Health Perspective

**DOI:** 10.1155/2016/2430945

**Published:** 2016-01-10

**Authors:** Sabrina Destri Emmerick Campos, Nathalie Costa da Cunha, Nádia Regina Pereira Almosny

**Affiliations:** ^1^Departamento de Patologia e Clínica Veterinária, Universidade Federal Fluminense, 24230-340 Niterói, RJ, Brazil; ^2^Departamento de Saúde Coletiva Veterinária e Saúde Pública, Universidade Federal Fluminense, 24230-340 Niterói, RJ, Brazil

## Abstract

There is increasing interaction between man and pathogens transmitted by arthropods, especially by ticks. It is on this background that a holistic approach stands out, for the sake of Public Health. Brazilian Spotted Fever is an endemic disease at the country's southeast, with* Amblyomma sculptum* as its major contributor, followed by* A. aureolatum* and potentially* Rhipicephalus sanguineus*. Dogs have been considered sentinels, and in some areas the disease in dogs can precede human disease. Considering the importance of this disease for human health, the serological evidence in dogs, and the transmission of ticks between dogs and their owners, this review aimed to elucidate the importance of the epidemiological investigation, the diagnosis in dogs, and the role of veterinarians in Public Health to control vector-borne zoonotic diseases. We encourage veterinarians to include this rickettsial infection in the diagnosis of febrile diseases of common occurrence in dogs.

## 1. Introduction 

Vector-borne diseases are globally important to human and animal health, since pathogens, vectors, and animal hosts reveal interactions through pathologies and their epidemiology, which differ among geographic zones, and may change over time [1]. Ticks and wildlife are among the main reservoirs of pathogens transmitted by arthropods of veterinary importance [2].

Human beings are causing important changes in the ecosystem, such as habitat fragmentation, global warming, and exploitation of natural resources, which have allowed interaction between man and pathogens potentially transmitted by arthropods [3, 4]. The global Strategic Framework for health has been created to decrease the risk and minimize the impact of emerging infectious diseases at the animal-human-ecosystem and socioeconomic interface [3]. Shaffer [5] suggested the use of surveillance policies for animals as part of an approach in One Health Perspective, in order to contribute to more coordinated actions towards human health.

It is against this background that stands out a holistic approach for the sake of Public Health, applying the concept of One Health, which recognizes that human welfare is linked to animals and the environment and so it seeks a combined action between physicians, ecologists, and veterinarians in the control of threats to Public Health.

*Rickettsia rickettsii* is the major bacterium responsible for the Brazilian Spotted Fever (BSF), a highly fatal disease with challenging diagnosis due to its nonspecific signs [6, 7]. To assist the epidemiological surveillance, studies have been searching for sentinel animals, such as horses and dogs, with positive serological reaction in endemic areas [8, 9].

Based on the importance of BSF to human health, the evidence of clinical illness in dogs from areas with laboratory-confirmed BSF in humans, and the potential transmission of ticks between dogs and their owners, this review aimed to discuss the importance of epidemiological surveys and laboratory diagnosis of BSF in dogs for Public Health, inspired by the principles of One World, One Health, a Strategic Framework that has been raised jointly by specialized agencies, such as World Health Organization (WHO), United Nations (UN) Food and Agriculture Organization (FAO), International Organization for Animal Health (OIE), and UN Children's Fund (UNICEF).

## 2. The One Health Idea 

The idea that humans, animals, and the ecosystems are closely related has been under discussion since the late nineteenth century, when the first movements to integrate activities and research in human and animal health were raised [3, 10].

It is believed that emerging infectious diseases are related to socioeconomic conditions and ecological features, which allow identifying potential hotspots of injuries of animal origin, particularly at low latitudes, at areas where the notifications are substantially weak [11], a pattern that fits the epidemiological situation of BSF.

The Strategic Framework establishes a more interdisciplinary approach with international cooperation, in order to ensure health for humans, animals, and ecosystems [3, 10, 12]. However, people are reservoir of only a small number of zoonotic pathogens, so it is understood that an effective monitoring system requires the integration of physicians, who can identify human outbreaks, and veterinarians, that can identify animal reservoirs and sentinels [5, 13].

Veterinarians can contribute to the promotion of health through their knowledge on environmental conservation, the use of domestic animals as sentinels for the circulation of pathogens in the domestic and/or wildlife arenas, and occupational risk (due to the exposure to ticks) [12, 14, 15]. A study with veterinary students revealed that animal treatment with acaricides, avoiding contact with ticks, keeping vegetation cut down, and inspecting the body every three hours for the presence of ticks were the main prevention methods cited in control of BSF [15].

We believe that interdisciplinary participation in epidemiological investigation research and dissemination of articles and reports to scientific and health care assistance communities could be a mechanism to integrate information and improve strategies to control this disease.

## 3. Brazilian Spotted Fever

### 3.1. History

A disease caused by* Rickettsia rickettsii* was first described in the USA, and since then it has been confirmed in several countries, including Canada, Mexico, Panama, Costa Rica, Colombia, Brazil, Argentina, and possibly Guatemala [16–21]. In Brazil, this disease has been called Brazilian Spotted Fever (BSF) and it was first discovered in the state of São Paulo in 1928, where it was originally treated as “Exanthematic Typhus” [22]. The return of BSF reports in the 80s has shown that the disease has never ceased to occur; however it became detected as an acute disease affecting people from the same household or labor, albeit isolated cases have been described [23, 24]. Since 2001, BSF is a nationally notifiable disease, considered endemic in southeastern Brazil [23, 25].

According to the Center of Disease Control, the gold standard serologic test for diagnosis of the disease is the indirect immunofluorescence assay (IFA) with* R. rickettsii* antigen, performed on two paired serum samples to demonstrate a significant (fourfold) rise in IgG antibody titers, since molecular diagnosis is not always routinely available to confirm cases (http://www.cdc.gov/rmsf/symptoms). In Brazil, 1141 human cases confirmed by IFA were notified between 2007 and 2015, being 61.26% (699/1141) in the southeast, especially in the states of São Paulo (43.21%, 493/1141), Minas Gerais (8.15%, 93/1141), and Rio de Janeiro (7.19%, 82/1141). The state of Santa Catarina is highlighted with 23.05% of human cases (263/1141). Amapá, Rondônia, and Amazonas contributed with a single confirmed human case each, in 2007, 2008, and 2011 (http://www.saude.gov.br/sinan).

### 3.2. Vectors, Reservoir, and Amplifying Hosts

Due to their capability to transmit a variety of zoonotic pathogens, ticks stand out on the concept of One Health. Brazil contains many biomes, rich and abundant fauna, and several species of arthropods, such as ticks of the* Amblyomma* genus, widely distributed in the Neotropical region [21].

Based on recent reassessment of the taxonomic and morphological status of* Amblyomma cajennense* (Fabricius, 1787), currently, the name* A. cajennense sensu lato *(*s.l.*) refers to a group of six species. According to geographical distributions, and host associations,* A. cajennense sensu stricto* (*s.s.*) applies to the tick found in the Amazonian region of South America, while* A. sculptum* applies to the tick found in the coastal states, and degraded areas of the Atlantic Forest, including all states in the southeast [26, 27].

Interestingly, the low host specificity of* A. cajennense s.l. *allows for their detection in many mammals including cattle, deer, and wild and domestic canids, besides man [7, 28, 29].* Amblyomma cajennense s.l.* is implicated as the major species responsible for BSF, followed by* A. aureolatum* [29, 30]. In addition, the potential disease-transmission role of* Rhipicephalus sanguineus* is also increasingly studied [9, 31–35].

In the tick, transstadial and transovarian systems maintain the bacteria, which are transmitted to the vertebrate host during blood feeding, making arthropods simultaneously vector and reservoir [36–39]. However, infection rates by* R. rickettsii* in ticks under natural conditions tend to be low (<1%), evidencing that* R. rickettsii* is pathogenic to ticks, and reinforcing the need of an amplifying host to ensure the maintenance of bacteria, such as capybaras, especially in endemic areas of São Paulo [40–44].

### 3.3. *Amblyomma sculptum*

In southeastern Brazil, the area with the highest concentration of BSF reports, the ecological setting in which the disease occurs is well described, including a voluminous population of* Amblyomma* ticks [21]. Horses and capybaras are among the most important primary hosts for all parasitic stages of* A. cajennense s.l. *[38, 45]. The capybara population has greatly increased in the state of São Paulo, and at this point it raises suspicions of its relationship to the emergence of BSF [44].

Although massive infestations with adult ticks occur in horses and capybaras in southeastern Brazil, nymphs of* A. cajennense s.l. *have shown better competence as vectors of* R. rickettsii* experimentally [46, 47], which is important, since these stages have less requirements regarding their hosts including dogs and people [28, 29]. In fact, most human cases of BSF seem to occur during the nymphs season of* A. cajennense s.l.*, from July to November, possibly related to the aggressive behavior of nymphs, their effective spreading through the environment, and their small size, making their removal quite difficult [48].

### 3.4. *Amblyomma aureolatum*

*Amblyomma aureolatum* is yet another vector involved in BSF, with its ecological peculiarities. The “yellow dog tick” is found mainly in subtropical areas, with high humidity and mild temperatures throughout the year [49]. The population of* A. aureolatum* tends to be low and in southeastern Brazil, its distribution is restricted to Atlantic Forest, typically occurring in dogs with free access to rainforest [30, 49].

There are few reports of adults of* A. aureolatum* biting humans [50, 51]. Thus, human cases transmitted by* A. aureolatum* seem to occur when dogs get infected by adults of this tick during incursions into the rainforest and go on to carry* A. aureolatum* to their households [21]. However, experimentally, this tick was more susceptible to* R. rickettsii* infection and more efficient to maintain the pathogen by transstadial and transovarian transmission than* A. sculptum *[52]. Another study with experimental infection of* A. aureolatum* demonstrated that* R. rickettsii* was preserved between transstadial and transovarial stages in 100% of the* A. aureolatum *ticks for several consecutive generations, and larvae, nymphs, and adults transmitted* R. rickettsii* to susceptible guinea pigs [53]. Recently it was suggested that the adult* A. aureolatum* needs only approximately 10 minutes attached to the body of a vertebrate host to transmit* R. rickettsii* [35].

Note that in some areas of southeastern Brazil, particularly in areas of the state of São Paulo,* A. aureolatum* can replace* A. sculptum* as the main vector of* R. rickettsii* to humans, being even more effective in transmitting the pathogen [30, 31, 53].

### 3.5. *Amblyomma ovale*

In the past years,* A. ovale* has been implicated as a possible vector of new rickettsiosis in Brazil, since a human case of febrile disease with eschar (*tache noire*) was observed after tick biting in an Atlantic (ATL) Rainforest region of the state of São Paulo. Phylogenetic analysis revealed a new human rickettsiosis of the Spotted Fever Group (SFG), named ATL Rainforest Rickettsiosis, distinct from that caused by* R. rickettsia* [54]. Due to its phylogenetic similarity to* R. parkeri*, this new strain was called* R. parkeri* strain ATL Rainforest, which has been detected naturally infecting* A. ovale* ticks [55, 56]. So far* A. triste* was the vector associated with infection by* R. parkeri* in South America, including Brazil [57, 58].

Experimentally, 100% of transstadial and transovarian transmission of* R. parkeri* to* A. ovale* was confirmed by PCR. Larvae and nymphs demonstrated high competence in transmission of the bacteria because all animals infested by these ticks' stages presented seroconversion when tested by IFA using* R. parkeri *antigens, but only half of the animals presented seroconversion after being infested by infected-adult ticks. Reproductive parameters of infected* A. ovale *females were low when compared to uninfected females, indicating deleterious effect of* R. parkeri* on this tick [56].

Amplifier hosts and reservoir in the ATL Rainforest Rickettsiosis have not been determined so far, but small rodents were shown to be important for* A. ovale* immature stages and high seroconversion prevalence [59]. Krawczak [56] suggested that* A. ovale* is an important vector on the epidemiology of* R. parkeri* strain ATL Rainforest, since this tick is often found in the ATL Rainforest ecosystem. Moreover, human bites by adult* A. ovale* tick are common [50]. Thus, humans can become infected if bitten by ticks detached from the dogs, suggesting the importance of dogs from the forests of endemic areas, mainly infested with* A. ovale* ticks [21, 59].

Serology studies have reported that dogs seroconvert, attaining very high titers against* R. parkeri* antigens [55, 59, 60]. Furthermore, Medeiros et al. [60] reported identical sequences to* R. parkeri* strain ATL Rainforest in dogs coinfested with* A. ovale* ticks. So far, it can be supposed that some febrile human cases diagnosed as mild BSF were in fact ATL Rainforest Rickettsiosis [59].

### 3.6. *Rhipicephalus sanguineus*

The “brown dog tick” is possibly the tick with greater distribution, inhabiting urban and rural environments where dogs and humans live [18]. It is a three-host tick that feeds primarily on dogs and occasionally on other hosts [61]. Participation of* Rh. sanguineus* in the transmission of BSF is still a source of speculation, although this tick is already an important vector and reservoir of* R. conorii* responsible for the Mediterranean Spotted Fever in Europe, Africa, and Asia [21].

In Brazil, human parasitism by* Rh. sanguineus* has been reported [61] but is still considered a rare event, particularly considering the close proximity of these ticks with man [51, 61]. Natural infection of* Rh. sanguineus *by* R. rickettsii* in Brazil has been observed in endemic areas for BSF [30, 33, 62]. And even if the transmission to humans has not been proven yet, there is a favorable outlook in urban areas where* Rh. sanguineus *is often found in pet or stray dogs [21]. Furthermore, these dogs can often move between urban and rural farming areas, being parasitized by ticks from both environments [30].

It is noteworthy that* Rh. sanguineus* tends to be less aggressive to man, making the transmission occasional, particularly for those dealing with dogs most of the time [21].

The traditional mechanism by which the tick gets infected by* R. rickettsia* is during blood feeding of the vertebrate host or by transstadial and transovarian transmission [36, 37]. However, under natural conditions, low infection rates among tick populations suggest that these mechanisms are not enough to maintain the pathogen in the ecosystem [40, 41]. It is important to consider that* R. rickettsii* is pathogenic to the tick, causing decreased fertility and death [41]. Cofeeding transmission of* R. rickettsii* is not fully elucidated but may have an important role in the transmission of bacteria among ticks that feed in close proximity at the same host [39].

Considering that dogs can become infected by* A. aureolatum* during incursions into the rainforest and that bacteria-infected* A. aureolatum* can remain on the dog for several weeks, it is possible that tick-infested dogs carry infected ticks back to human households. The ticks may then drop off from the dog, contaminate the household environment, and accidentally bite humans. In a different scenario,* Rh. sanguineus* ticks can become infected by cofeeding on the same dog with infected* A. aureolatum* ticks [21, 49, 59].

## 4. *Rickettsia rickettsii* Infection in Dogs and the Role of Sentinels in Brazil

Clinical disease caused by* R. rickettsii* in dogs is not easy to diagnose and has not been well described in South America, with few cases reported in the state of São Paulo, Brazil [7, 63].

Experimentally, dogs developed clinical illness characterized by fever, lethargy, anorexia, bilateral ocular discharge, scleral congestion, conjunctival edema, thrombocytopenia, and anemia [64].* Rickettsia rickettsii*-reactive antibodies were shown in serum samples, and rickettsial DNA was detectable in blood 3 to 13 days after infection, indicating that a Brazilian strain of* R. rickettsii* is pathogenic for dogs [64].

Dogs, which remain close to both humans and naturally infected areas, can play a role as sentinels in an epidemiological approach [34, 65]. The use of serological methods for the detection of anti-*Rickettsia* spp. antibodies in dogs has been reported in several Brazilian states, especially in the southeast [9, 32, 34, 62, 66].

According to Cunha et al. [34], the dog's habit of entering rainforest regions and living in rural environments indicated a risk factor to the presence of anti-SFG rickettsiae antibodies.

Although SFG species share antigens that might cause group reactive serological responses, IFA is a highly sensitive and specific technique, used as the method of choice in serosurvey and screening tests in Brazil [67, 68]. In addition, if serology to several SFG antigens demonstrates titers to one antigen at least fourfold higher than the others, we can assume which pathogen stimulated the immune response [9]. High titers to* R. rickettsii* in endemic areas for BSF, up to 1 : 4096, or more, reinforce this hypothesis [34].

Clinical signs in dogs and humans may be similar, in such a way that the disease in dogs can precede the disease in humans, reinforcing the role of dogs as sentinels of BSF, a hypothesis that becomes stronger in USA, where cases of* R. rickettsii* in dogs and their owners have been found [8, 69, 70].

In Brazil, canine monocytic ehrlichiosis (CME), caused by* Ehrlichia canis*, is the most common tick-transmitted canine disease [71]. As many clinical and laboratorial findings described in CME, fever, depression, petechial hemorrhage, and thrombocytopenia, are also described in dogs infected by* R. rickettsii* [72], and given that doxycycline is the treatment of choice for both diseases, it is possible that rickettsial infection in dogs is being misdiagnosed as CME [7, 9, 72].

Differential diagnosis is a great challenge, because dogs may present with subclinical infections or nonspecific clinical signs, often misdiagnosed as other diseases transmitted by arthropods like CME, Lyme's disease, babesiosis, leishmaniasis, anaplasmosis, and any febrile disease of unspecified etiology [73].

According to Labruna et al. [7], definitive diagnosis of naturally infected dogs is based on (1) serological analysis of paired samples; (2) anti-*R. rickettsii* titers fourfold higher than other spotted-fever group antigens occurring in Brazil; (3) rickettsial DNA detection in blood; (4) both clinical and laboratorial findings compatible with the disease; (5) doxycycline responsive treatment; and (6) epidemiological history with tick infestation and exposure to endemic areas.

Besides the dog, authors have attempted to elucidate the role of other domestic animals as sentinels of the BSF. Horses, considered primary hosts of* A. cajennense s.l.*, are also an important object of study in the epidemiology of the disease.

Participation of horses as sentinels of infections caused by SFG has also been reported. One study suggested the ecological importance of cart horses as sentinels for BSF, since these horses are extensively used for transporting humans and heavy loads in urban and rural areas, being heavily infested with ticks [74]. Vianna et al. [75] believed that horses could be better sentinels to* R. rickettsii* than dogs, due to the presence of antibodies anti-*R. rickettsii* in 100% of the equine sera tested by IFA. However, Cunha et al. [34] after serosurvey in human foci of BSF observed that if the vector is not* A. sculptum*, horses present low serological reaction rate, which rules out these animals as good sentinels, while dogs can perform the role of sentinel for different vectors, since they can be parasitized by* A. sculptum*,* A. aureolatum*,* A. ovale*, and* Rh. sanguineus*. This was reinforced by other studies in the state of São Paulo, which observed a higher frequency of horses with positive serology in areas with strong evidence that the main vector was* A. cajennense s.l.* [66].

## 5. Conclusion and Perspectives

Ticks of domestic animals may be involved in the epidemiology of several vector-borne diseases, which also affect humans. Even though the dogs are not the main host for* R. rickettsii*, they may carry infected ticks into the human dwellings. One of the most effective ways to assess the evidence of SFG pathogens circulation in sentinel animals is through serological tests, among which the IFA has been widely employed in dogs, particularly because of the easy access to samples, their intimate relationship with man, and parasitism by the same ticks.

Due to this challenging outlook it is possible that BSF in dogs is underestimated, since the nonspecific clinical signs may get confused with EMC, which is the most prevalent tick-borne disease in dogs. As doxycycline is an effective treatment for patients with BSF or EMC, it is possible that dogs with acute febrile illness are not being routinely molecularly tested for BSF, since its cost is a limiting factor. Veterinarians should include rickettsial infections in the differential diagnosis of CME and other febrile diseases of nonspecific signs transmitted by common ticks, facilitating monitoring of BSF, since it is an important zoonosis with human fatalities when the diagnosis is delayed and treatment cannot be implemented in time.

Serology of these dogs could indicate prior exposure to rickettsial agents by the presence of IgG antibodies even before the reporting of human cases of BSF, warning about circulation of the bacteria, which added to the knowledge of the presence of ticks could help to improve BSF monitoring.

When physicians, veterinarians, and other health professionals face every challenge the same way, they will understand that the traditional mechanisms for the study of diseases are full of unresolved gaps that can be addressed with interdisciplinary actions.
